# Hypofractionated Stereotactic Radiotherapy for the Treatment of Benign Intracranial Meningiomas: Long-Term Safety and Efficacy

**DOI:** 10.3390/curroncol28050314

**Published:** 2021-09-23

**Authors:** Eric K. Nguyen, Gregory R. Pond, Jeffrey N. Greenspoon, Anthony C. Whitton, Crystal Hann

**Affiliations:** 1 Division of Radiation Oncology, Juravinski Cancer Center, Hamilton, ON L8V 5C2, Canada; eric.nguyen@medportal.ca (E.K.N.); greenspj@hhsc.ca (J.N.G.); whittona@HHSC.CA (A.C.W.); 2 Department of Oncology, Escarpment Cancer Research Institute, McMaster University, Hamilton, ON L8S 4L8, Canada; gpond@mcmaster.ca

**Keywords:** meningiomas, radiosurgery, SRS, hypofractionation, benign, intracranial

## Abstract

Introduction: Hypofractionated stereotactic radiotherapy (hSRT) has emerged as an alternative to single-fraction stereotactic radiosurgery (SRS) and conventionally fractionated radiotherapy for the treatment of intracranial meningiomas (ICMs). However, there is a need for data showing long-term efficacy and complication rates, particularly for larger tumors in sensitive locations. Methods: A retrospective review was conducted on adult patients with ICMs seen at a tertiary care center. Eligible patients were treated with the CyberKnife platform and had a planned treatment course of 3–5 fractions from 2011–2020. The local control was assessed based on radiographic stability and the late toxicity/radionecrosis rates were recorded. Radiographic progression-free survival (PFS) and overall survival (OS) were estimated using the Kaplan–Meier method. Results: In total, 62 patients (age 26–87) with 67 treated tumors were included in this study with a median follow-up of 64.7 months. RT was delivered as the primary treatment in 62.7% of cases and for recurrence in 37.3%. The most common tumor locations were the convexity of the brain and the base of the skull. The tumor sizes ranged from 0.1–51.8 cc and the median planning target volume was 4.9 cc. The most common treatment schedule was 18 Gy in 3 fractions. The five-year PFS and OS were 85.2% and 91.0%, respectively. The late grade III/IV toxicity rate was 3.2% and the radionecrosis rate was 4.8%. Conclusions: Based on our data, hSRT remains an effective modality to treat low-grade ICMs with acceptable long-term toxicity and radionecrosis rates. hSRT should be offered to patients who are not ideal candidates for SRS while preserving the benefits of hypofractionation.

## 1. Introduction

Intracranial meningiomas (ICMs) are the most common central nervous system tumor in adults, comprising 20–30% of all primary brain tumors [1,2]. They arise from the arachnoid layer of the meninges between the dura mater and pia mater, commonly developing at sites with a high density of arachnoid villi [2]. The majority of these lesions are considered to be benign with approximately 90% being classified as Grade I under the World Health Organization (WHO) grading system [3,4]. These exhibit a relatively slow growth with a lower mitotic activity and necrosis compared with higher graded tumors but may require a definitive treatment, particularly if they encroach on critical structures and cause significant neurological symptoms [5].

For ICMs that necessitate treatment, the gold standard is maximal safe surgical resection with the extent of resection determining the post-surgical approach. However, for tumors that are in surgically inaccessible or risky locations, or for patients that are generally poor surgical candidates, external beam radiotherapy is often considered as an upfront option [6,7]. Conventionally fractionated radiotherapy (cRT) typically consists of several weeks of a fractionated treatment, but with the advent of improved patient immobilization with high-precision image guidance, stereotactic approaches have become much more prominent. Single-fraction stereotactic radiosurgery (SRS) has emerged as an effective and more convenient alternative for select patients, taking into account the tumor location and proximity to sensitive neuroanatomy [8,9]. SRS has been shown to have excellent local control rates for ICMs and is most effective for smaller lesions that are situated at a safe distance from the optic pathways and other critical structures [10].

Hypofractionated stereotactic radiotherapy (hSRT) is becoming increasingly used for ICMs in modern practice, establishing a prominent role alongside SRS and cRT. hSRT retains the radiobiological advantages of fractionation while achieving higher doses per fraction over a shorter timeframe compared with a conventional treatment. This may present benefits for treating larger tumors and those located close to sensitive structures that would not be amenable to SRS.

Despite its potential, there is limited high-level evidence supporting the use of hSRT for ICMs. Retrospective data have accumulated but there is a need for larger studies with extended outcomes. Here, we report on a modern cohort of Grade I ICM patients treated with hSRT to assess the tolerability and long-term control of this modality. 

## 2. Methods

A retrospective review was conducted on adult patients with Grade I ICMs treated at the Juravinski Cancer Center (JCC) between 1 January 2011 and 31 December 2020. The JCC is a tertiary care center in Hamilton, Ontario, Canada, covering a local health integration network with a catchment of approximately 1.4 million people. Eligible patients were treated using a stereotactic approach and received a planned treatment course of 3–5 fractions for primary or recurrent disease. Patients were required to have a surveillance MRI and a follow-up visit at least 3 months following the treatment.

Procedures were in accordance with the ethical standards of the Helsinki Declaration of the World Medical Association. The design of the study was approved by the local institutional ethics board (Hamilton Integrated Research Ethics Board). 

All patients were treated on the CyberKnife platform (Accuray, Inc., Sunnyvale, CA, USA). Patients underwent planning and treatment with an aquaplast cast and a cantilever board used for immobilization. The CT simulation slice thickness was 1 mm and an MRI simulation with gadolinium was performed in all cases. Pre-treatment diagnostic imaging was fused to the planning scans as appropriate. The gross tumor volume was delineated as the enhancing lesion on the MRI T1 sequence with a gadolinium contrast. The planning target volume was 1 mm as per the institutional standard for CyberKnife.

Data were abstracted from patient charts by a primary author (EN) including demographics, lesion size, location, histological details, and pre-treatment symptoms. Treatment data included radiotherapy dose-fractionation, treatment volume, prescription isodose line, and the conformity/homogeneity index. The local control was assessed based on radiographic stability using the Response Assessment in Neuro-Oncology (RANO) criteria [11]. Late toxicity was recorded based on the Common Terminology Criteria for Adverse Events, version 5. Radionecrosis was assessed based on a combination of radiographic findings and documented clinical suspicion. The primary outcome was radiographic progression-free survival (PFS) and the secondary outcomes included overall survival (OS), local control, late Grade III/IV toxicity, and radionecrosis rates.

Descriptive statistics were used to summarize the patient characteristics and outcomes. The Kaplan–Meier method was used to estimate the time-to-event outcomes including radiographic PFS (defined as the date of the diagnosis to relapse or death due to any cause) and overall survival (OS, defined as the date from the diagnosis to death due to any cause). Cumulative incidence methods were used to estimate the local control (defined as the date from the diagnosis to radiographic progression) rates and non-cancer-related deaths were considered a competing risk. Patients without an outcome event were censored at the final follow-up. A Cox proportional hazards regression was performed to investigate the factors potentially prognostic of the outcomes including the use of the cause-specific hazard function for the local control. Forward stepwise selection was conducted to construct a multi-variable model. The conformity index (CIN) and the new conformity index were highly non-normal so the data were dichotomized into two groups at the median value for statistical purposes. Due to small numbers, only the results of the composite outcome—the radiographic PFS—are presented in detail. All tests were two-sided and a *p*-value of <0.05 was considered statistically significant. 

## 3. Results

A total of 62 patients with an age ranging from 26 to 87 (median = 59) with 67 treated tumors were included in this study. Overall, 84 charts were reviewed with 22 patients being excluded; 15 had a higher grade histology, 5 were lost to follow-up prior to the reassessment, and 2 were treated but did not have their 3-month follow-up. Primary RT was delivered in 38 (61.3%) cases and RT following a recurrence was delivered in 24 (38.7%). The most common tumor locations were the convexity of the brain in 27 (40.3%) patients and the base of the skull in 22 (32.8%) (Table 1). Overall, 43 (69.4%) patients had pre-treatment symptoms thought to be clinically related to the treated ICM that had prompted the therapy, 15 (24.2%) patients were treated for an asymptomatic tumor growth, and 4 (6.4%) were treated on the basis of the tumor location alone.

Table 2 lists the tumor and treatment details. The tumor size ranged from 0.1 to 51.8 cc with a median of 4.9 cc. The median PTV volume was 7.5 cm^3^ and the median prescription isodose line was 75%. The total dose ranged from 14 to 25 Gy in 3 to 5 fractions with the most common schedule being 18 Gy in 3 fractions (35.8%). The dose-fractionation was determined based on standardized institutional guidelines that recommend 18 Gy in 3 fractions for a PTV of 3.0–3.9 cm and 25 Gy in 5 fractions for a PTV ≥ 4.0 cm. The decision to treat with hSRT based on the tumor location alone was made by the treating clinician. The treatment was completed as planned in 98.4% of patients with one patient stopping therapy early due to uncontrolled pseudoseizures. 

After a median follow-up of 64.7 months, the crude local control rate was 94.0% amongst the 67 lesions with 54 (80.6%) lesions having a stable radiographic response and 9 (13.4%) having an interval decrease in size. In total, 6% of the treated lesions had a radiographic progression with a median of 34.9 months from the completion of the treatment to the date of the reported progression. 

**Figure 1:**
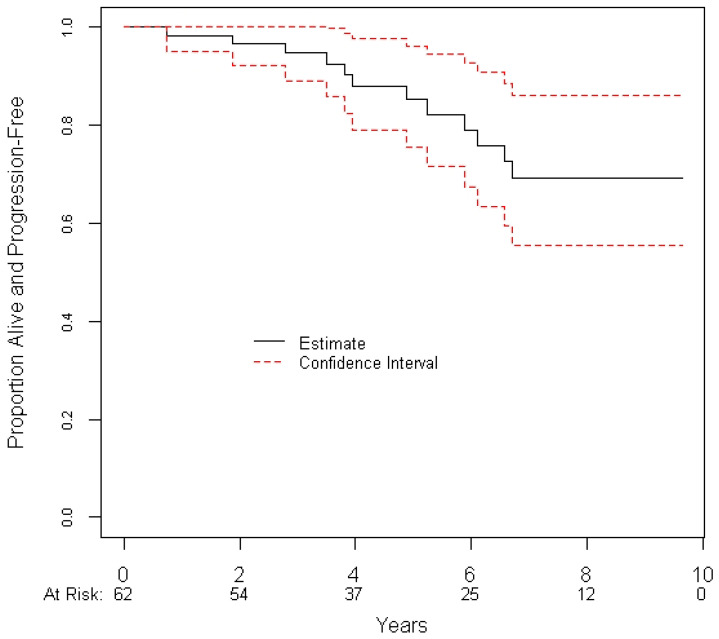
Progression-free survival of patients treated with hSRT.

Amongst the patients, 49 (79.0%) were stable and 9 (14.5%) had a radiographic response. Four (6.4%) patients had a radiographic progression, of whom 1 patient had a re-resection, fractionated radiation and systemic therapy; 2 patients had systemic therapy alone; and 1 patient had a re-resection alone. All four patients were alive at the final follow-up; however, 8 (12.9%) other patients died without having progressed radiographically. Kaplan–Meier estimates for the 5-year radiographic PFS (Figure 1) and 5-year OS were 85.2% (95% confidence interval (CI) = 71.1% to 92.8%) and 91.0% (95% CI = 77.6% to 96.6%), respectively. The 5-year local control rate was 94.4% (95% CI = 100% to 87.7%). 

Two (3.2%) patients experienced late Grade III/IV toxicities and 3 (4.8%) patients experienced radionecrosis: two cases were symptomatic, one patient was managed with a surgical resection and the second was managed conservatively with corticosteroids. Radionecrosis occurred at 2.6 months for 2 patients and 7.4 months for the third patient. There were no deaths attributable to ICMs or treatment-related complications.

**Figure 2:**
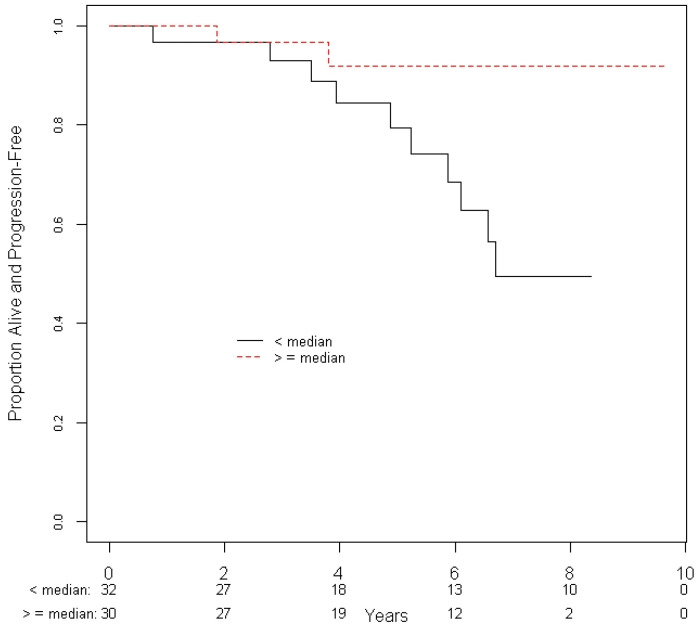
Progression-free survival of patients based on the conformity index in relation to the median values of the cohort.

Based on univariate analyses, a higher CIN was associated with an improved PFS (hazard ratio = 0.19, 95% CI = 0.04 to 0.86 for > median vs. < = median, *p* = 0.032, Table 3). Of the 32 patients with a CIN greater than the median of 1.13, 10 patients had a recurrence or died; of those with a CIN less than or equal to the median, only 2 patients had a recurrence or died (Figure 2). There were no other prognostic factors that had a significant association with PFS.

## 4. Discussion

In this single-institution retrospective analysis, we report the modern outcomes of patients with Grade I ICMs treated with hSRT using the CyberKnife platform. The 5-year radiographic PFS and OS were 85.2% and 91.0%, respectively, with a 5-year local control rate of 94.4%. The treatment was well-tolerated with a late Grade III/IV toxicity rate of 3.2% and a radionecrosis rate of 4.8%. This study shows the long-term efficacy of hSRT in a large cohort of benign ICM patients with low rates of toxicity even when treating larger tumors or those located in sensitive locations.

The use of single-fraction SRS for ICMs has been well-established in the literature. Santacroce et al. published a multi-center review of 4565 patients with ICMs treated with SRS and reported 5-year and 10-year PFS rates of 95.2% and 88.6%, respectively [8]. Permanent morbidity occurred in 6.6% of patients. In addition, Chung et al. completed a systematic review of ICM studies and, in a comparison with cRT, found that SRS had a similar efficacy with a 5-year PFS of 93.2%. The average total complication rate was 9.2% [12]. Although SRS has been shown to be safe and efficacious for smaller, well-localized ICMs, tumors that are larger in size or located outside of the skull-base regions may have a higher risk of complications when using single-fraction approaches. Pollock et al. described a significantly higher rate of toxicity in larger tumor volumes that were greater than 9.6 cc with a radiation-related complication rate of 22.6% compared with 4.8% in smaller lesions [13]. In addition, Sheehan et al. reported that 38.2% of patients receiving SRS for parafalcine or parasagittal ICMs developed new or worsened perilesional swelling with tumor size and a venous sinus invasion being predictive factors for a post-SRS edema [14]. 

The CyberKnife platform is a frameless robotic radiotherapy unit that delivers stereotactic radiation using inverse planning. It utilizes a 6 MV linear accelerator mounted to a robotic arm with 6 degrees of freedom and real-time X-ray image guidance [15]. The radiation dose is delivered at a number of nodal positions defined in a sphere around the patient, which can be non-isocentric from hundreds of non-coplanar angles, allowing for a higher degree of dose drop-off compared with a linac-based SRS. Furthermore, unlike other SRS platforms such as GammaKnife, CyberKnife uses a multi-leaf collimator to block a broad beam and the treatment length is not dependent on the age of the source, minimizing the beam-on time [16]. This provides advantages as a platform for SRS and allows for a more efficient treatment, particularly when delivering multi-fraction stereotactic courses.

The emergence of hSRT presents a compelling alternative to SRS that maintains the convenience of limited fraction treatment courses whilst providing a broader scope of application. A recent systematic review showed promising results in regard to the efficacy of hSRT in an ICM treatment [17]. Although the majority of the publications had small patient cohorts, the crude local control was 90–100% as reported in 10 studies and the median 5-year PFS was 88% as reported in 4 studies. Notable reports include Han et al. who compared hSRT with both SRS and cRT for the upfront treatment of ICMs and found no significant difference in the clinical response, late toxicities, or PFS [18]. In addition, Albert et al. demonstrated a trend toward improved 3-year OS in post-operative patients receiving SRS or hSRT compared with cRT [19]. 

In the current study, the 5-year PFS was 85.2%, which is similar to data in the published literature and comparable with the rates for SRS and cRT in historical reports [8,12]. There was no difference in PFS or late toxicity when comparing 3-fraction and 5-fraction regimens although 88.9% of those who had a significant radiographic response received 25 Gy in 5 fractions with a mean lesion size of 15.2 cc in this cohort. Whether the increased biological effective dose inherent to the 5-fraction treatment schedule played a factor in the response may be considered but further supports the use of extended hSRT courses, particularly for larger lesions.

In terms of the treatment tolerability of hSRT, Nguyen et al. reported a median late toxicity rate of 8% with a range of 0% to 21% across 12 studies [17]. The most common late toxicities were a decreased visual acuity and a new cranial neuropathy with a Grade 3 or higher toxicity reported in a total of 3 patients. hSRT appears to have fewer restrictions in regard to the ICM location and, as evidenced by Colombo et al., patients with lesions close to critical structures had low rates of toxicities when treated with hSRT including patients that could not have been treated with SRS otherwise [20]. Similarly, Girvigian et al. reported on ICMs treated in the brain convexity or parasagittal regions and described symptomatic edema rates of 6.3% with hSRT compared with 43% using SRS [21]. The present study showed late Grade III/IV toxicity rates to be low, demonstrating safety when treating ICMs located in the skull base, convexity, or parasagittal/parafalcine regions. One patient had significant confusion and unilateral weakness related to necrosis that required surgical intervention but ultimately survived.

Based on the univariate analyses, a higher CIN was associated with an improved PFS and more patients had recurrences or died when the CIN was above the median. This is contrary to the classical opinion that a CIN closer to 1.0 is indicative of an optimal plan that maximizes the local control and minimizes toxicity [22,23]. This may be explained by several factors inherent to the nature of CIN and the relationship with the lesion size and shape. In general, larger lesions can result in smaller conformity values with a combination of over-coverage and under-coverage in separate regions, presenting the impression that the plan is of a higher quality with a lower CIN [24]. Furthermore, as described by Mansouri et al., CIN does not take into account the quality of the coverage, particularly when treating lesions that are asymmetrical and non-spherical [25]. With ICMs, the coverage of the dural tail may result in a higher CIN, sacrificing improved conformality for potential benefits in the local control although this is still an area of debate [26]. Nevertheless, although CIN can be used in a stereotactic treatment evaluation, it is only one component of the plan quality that should be considered within the specific clinical context.

This study had a number of limitations including its retrospective design and heterogeneous population. Data were reported from a single center at a tertiary care clinic and the results may not be generalizable to other centers with differences in practice. Clinical and treatment data were based on the dictated reports available and this restricted the ability to fully assess symptoms, toxicity, and clinical responses in the follow-up. Furthermore, although the authors used the RANO criteria to assess the radiographic stability of lesions, when imaging was not available, they relied on dictated notes alone and the associated variance in the measurement approach depended on the radiologist. Finally, although the sample size was larger than most current reports on this topic, there was a limited overall power, especially to detect moderate or small effects.

## 5. Conclusions

In conclusion, our retrospective study supports the use of hSRT for low-grade ICMs in upfront and recurrent settings. The long-term follow-up showed an excellent PFS with low rates of complications even in more sensitive tumor locations. We recommend the use of hSRT for the definitive treatment of ICMs, especially for larger lesions and those situated in close proximity to critical structures. 

## Figures and Tables

**Table 1:** Descriptive data.

Characteristic	Units	Value
Age at Treatment	Median (range)	59 (26, 87)
Lesion Type	N (%) Primary	38 (61.3)
Prior Meningiomas	N (%) 1	5 (8.1)
Multiple Lesions	N (%) Yes	11 (17.7)
Perilesional Edema	N (%) Yes	5 (8.1)
Lesion Location Base of Skull Convexity Parafalcine/Parasagittal Other	N (%)	22 (32.8) 27 (40.3) 15 (22.4) 3 (4.5)
Simpson Resection Grade 1 2 3 4	N (%)	0 9 (42.9) 0 12 (57.1)
Pre-treatment Symptoms Diplopia Headache Headache with proptosis Headache with seizures Seizures Tremors Trigeminal Neuralgia Vertigo Vision Issues Visual Disturbance Hearing Loss, Facial Numbness Seizures	N (%)	6 (14.0) 18 (41.9) 1 (2.3) 2 (4.7) 3 (7.0) 1 (2.3) 6 (14.0) 2 (4.7) 1 (2.3) 1 (1.3) 1 (1.3) 1 (2.3)

**Table 2:** Treatment details.

Characteristic	Median (Range)
Size (cc)	4.9 (0.1, 51.8)
PTV (mm^3^)	7492 (534, 37939)
Prescription Isodose	75 (65, 90)
Maximum Dose	2769 (2118, 3731)
Conformity Index	1.13 (1.00, 3.51)
Normalized Conformity Index	1.16 (1.04, 3.63)
Homogeneity Index	1.33 (1.18, 1.54)
Dose	N (%)
18 Gy/3 Fractions 20 Gy/5 Fractions 21 Gy/3 Fractions 25 Gy/5 Fractions	32 (47.8) 1 (1.5) 3 (4.5) 31 (46.3)

**Table 3:** Prognostic factors of radiographic progression-free survival using a Cox proportional hazards regression.

Characteristic	Statistic	N	Hazard Ratio (95% CI)	*p*-Value
Age at CyberKnife Treatment	/Year	62	1.01 (0.97, 1.07)	0.59
Lesion Type	Primary vs. Recurrence	62	0.98 (0.30, 3.27)	0.98
Location	Parafalcine/Parasagittal Base of Skull Convexity Other	62	Reference 0.74 (0.12, 4.44) 1.26 (0.25, 6.31) 1.88 (0.17, 20.94)	0.83
Multiple Lesions	N vs. Y	62	2.16 (0.28, 16.71)	0.46
Perilesional Edema	N vs. Y	62	0.58 (0.13, 2.66)	0.48
First diagnosis to CyberKnife	/Month	48	0.93 (0.61, 1.43)	0.75
Prior Resection	Y vs. N	62	1.19 (0.36, 3.96)	0.78
Prior Recurrence	Y vs. N	62	1.01 (0.30, 3.36)	0.99
Size (cc)	/Log-Unit	62	1.43 (0.84, 2.44)	0.19
PTV (mm^3^)	/Log-Unit	62	2.17 (0.97, 4.84)	0.058
Prescription Isodose	/Unit	62	0.95 (0.86, 1.06)	0.38
Maximum Dose	/100 Units	62	1.12 (1.00, 1.26)	0.052
Conformity Index	>Median vs. < = Median	62	0.19 (0.04, 0.86)	0.032
New Conformity Index	>Median vs. < = Median	62	0.34 (0.09, 1.26)	0.11
Homogeneity Index	/0.01 Units	62	1.05 (0.99, 1.13)	0.13
Dose	/100 Units	62	1.15 (0.96, 1.38)	0.12
Fractions	3 vs. 5	62	0.34 (0.10, 1.15)	0.083
Multi-variable Results				
Conformity Index	/0.01 units	62	0.92 (0.84, 1.00)	0.047

## Data Availability

The datasets generated during and/or analyzed during the current study are available from the corresponding author on reasonable request.
